# Endovascular thrombectomy in acute ischemic stroke patients with prestroke disability (mRS ≥2): A systematic review and meta-analysis

**DOI:** 10.3389/fneur.2022.971399

**Published:** 2022-09-15

**Authors:** Jin-Cai Yang, Qiang-Ji Bao, Yu Guo, Shu-Jun Chen, Jin-Tao Zhang, Qiang Zhang, Ping Zhou, Ming-Fei Yang

**Affiliations:** ^1^Graduate School, Qinghai University, Xining, China; ^2^Department of Neurosurgery, Qinghai Provincial People's Hospital, Xining, China

**Keywords:** ischemic stroke, disability, thrombectomy, outcomes, meta-analysis

## Abstract

**Objective:**

The effect of endovascular thrombectomy (EVT) in acute ischemic stroke patients with prestroke disability (modified Rankin Scale score, mRS) ≥2) has not been well-studied. This study aimed to assess the safety and benefit of EVT in patients with prestroke disability.

**Methods:**

According to PRISMA guidelines, literature searching was performed using PubMed, Embase, and Cochrane databases, for a series of acute ischemic stroke patients with prestroke mRS ≥2 treated by EVT. Random-effects meta-analysis was used to pool the rate of return to prestroke mRS and mortality at 3-month follow-up.

**Results:**

In total, 13 observational studies, with 2,625 patients, were analyzed. The rates of return to prestroke mRS in patients with prestroke mRS of 2–4 were 20% (120/588), 27% (218/827), and 31% (34/108), respectively. Patients with prestroke disability treated by EVT had a higher likelihood of return to prestroke mRS (relative risk, RR, 1.86; 95% CI 1.28–2.70) and a lower likelihood of mortality (RR 0.75; 95%CI 0.58–0.97) compared with patients with standard medical treatment. Successful recanalization (Thrombolysis in Cerebral Infarction grade 2b-3) after EVT gave a higher likelihood of return to prestroke mRS (RR 2.04; 95% CI 1.17–3.55) and lower mortality (RR 0.72; 95% CI 0.62–0.84) compared with unsuccessful reperfusion.

**Conclusions:**

Acute ischemic stroke patients with prestroke disability may benefit from EVT. Withholding EVT on the sole ground of prestroke disabilities may not be justified.

**Systematic Review Registration:** https://www.crd.york.ac.uk/prospero/.

## Introduction

Current guidelines for acute ischemic stroke treatment unanimously recommend endovascular thrombectomy (EVT) for eligible patients with a prestroke modified Rankin Scale score (mRS) of 0–1 (1–3). The challenge of the available evidence from randomized clinical trials is partly due to the trial selection paradigms that exclude patients with prestroke mRS ≥2 (4). However, prestroke mRS ≥2 is relatively common among patients harboring acute ischemic stroke, with a reported frequency between 23.5 and 34.1% (4, 5). Furthermore, an international survey has reported that the EVT practice for patients with prestroke mRS ≥2 is heterogenous, and the EVT decision largely depends on clinician opinions (6). Therefore, selecting optimal treatments is necessary for these patients to obtain timely and successful revascularizations and improved clinical outcomes.

Beyond the previously published literature review, which has been limited to a broad overview of the current evidence (7), two systematic reviews of observational studies compare the outcome of EVT in patients with prestroke mRS ≥3 to those with mRS <3 (8, 9). Adamou et al. (8) have concluded that prestroke mRS ≥3 represents an independent predictor for unfavorable clinical outcomes. Bala et al. (9) have revealed that although patients with prestroke mRS ≥3 are related to an increased risk of death, higher proportions of patients reached their prestroke mRS. Because those systematic reviews categorize patients with prestroke mRS = 2 into the disability-free group, and only <15% of the patients in the analysis have prestroke mRS ≥3, concerns arise about selection bias and limited generalizability of the results. Moreover, no stratified analyses have been performed in the previous systematic reviews based on prestroke mRS categories (i.e., mRS 2, 3, and so on), and consequently, no effects of the disability degree on outcomes have been studied. In addition, the superiority of EVT and standard medical treatments (including intravenous thrombolysis, systematic anti-coagulation, antiplatelet medications, or combinations of these medical treatments) have not been well-evaluated. The benefit of successful reperfusion as a proxy for EVT is yet to be assessed.

The present systematic review and meta-analysis aim to (1) estimate the rate of differential outcomes of EVT in patients with prestroke disability, stratified by prestroke mRS; (2) assess the safety and efficacy of EVT in treating patients with prestroke disability, in comparison with standard medical treatment; and (3) evaluate the safety and benefit of successful recanalization achieved by EVT in patients with prestroke disability.

## Methods

The study protocol was prospectively registered in the PROSPERO registry (Registration No.: CRD42022327983). This systematic review and meta-analysis was conducted in accordance with the PRISMA statement (10) and was reported in compliance with the MOOSE guidelines (11).

### Eligibility criteria

Types of studies: prospective or retrospective observational studies. Types of participants: acute ischemic stroke patients with prestroke mRS ≥2. Types of interventions: EVT and /or standard medical treatment. Types of outcome measures: outcomes included a return to prestroke mRS and mortality at a 3-month follow-up.

### Search strategy

Systematic literature searching was conducted on Pubmed, Embase, and the Cochrane Library, from their inception to March 28, 2022, without any restrictions. Additional manual searching included the reference lists of all included studies and relevant review articles.

Complete searching keywords were as follows: ((“prestroke” OR “pre-stroke” OR (“stroke” AND (“premorbid” OR “pre-morbid” OR “preexisting” OR “pre-existing” OR “previous” OR “baseline”))) AND (“morbidity” OR “mobility impairment” OR “disability” OR “disabilities” OR “dependence” OR “dependent” OR “dependency”)) AND (“reperfusion therapies” OR “reperfusion treatments” OR “endovascular therapy” OR “endovascular treatment” OR “intra-arterial therapy” OR “intra-arterial treatment” OR “endovascular thrombectomy” OR “mechanical thrombectomy” OR “intra-arterial thrombectomy” OR “MT” OR “EVT” OR “IAT”). Our search was last updated on 3 June 2020 to ensure there were no new studies meeting the eligibility criteria.

### Study selection

The records obtained from electronic database searching were imported into the Zotero reference management software (www.zotero.org), and duplicates were removed. Two reviewers independently screened the titles and abstracts of the records for eligibility. Subsequently, all studies deemed eligible according to title and abstract screening were subjected to a full-text review by two independent reviewers. In the case of disagreements about the literature search results, the senior author (M-FY) was consulted to formulate a mutual consensus.

### Data extraction

Two reviewers independently extracted the data using a standardized template adapted from the Cochrane Collaboration. Information was collected on study characteristics (first author, year of publication, study period, country of origin, study design, number of institutions, included population, and number of patients), patient characteristics (age, sex, National Institutes of Health Stroke Scale, NIHSS, and Alberta Stroke Program Early CT Score, ASPECTS), and clinical outcomes. When duplicate reports of the same study were found, data from the most complete data set was analyzed. Disagreements were adjudicated by the senior author (M-FY).

### Risk of bias assessment

Two reviewers independently assessed the quality of observational studies included in this meta-analysis using the Newcastle-Ottawa Scale (NOS) (12). All these studies were reviewed and scored based on the following domains: selection of study groups (0–4 scores), comparability (0–2 scores), and assessment of outcomes (0–3 scores). A maximum number of nine scores could be awarded. Score ≥8 suggested a low risk of bias, 6–7 suggested a moderate risk of bias, and ≤5 suggested a high risk of bias. The potential disagreements were resolved through discussion with the senior author (M-FY).

### Statistical analysis

Stratified by prestroke mRS (mRS 2, 3, 4, and 5), the cumulative percentage and 95% CI for each outcome in patients treated with EVT were evaluated from each cohort. In addition, the effect (risk ratio RR with associated 95% CI) of EVT on outcomes in patients with prestroke disability was studied by meta-analyzing the rates in studies reporting data in patients treated with EVT vs. standard medical treatment. Furthermore, the effect estimates of outcomes were computed by analyzing the event rates in studies reporting data in EVT patients with successful recanalization vs. those without. Random-effect models with the inverse-variance method were used to combine studies to yield the overall effect (13). Statistical significance was determined using the equivalent *Z* test, with a 2-tailed value of *p* < 0.05 considered as the significance threshold. Heterogeneity among studies was assessed by the Cochran *Q* test at a significance level of *p* < 0.1 and quantified by the *I*^2^ statistic. *I*^2^-value < 50, 50% ≤ *I*^2^-value ≤ 75%, and *I*^2^-value > 75% were considered to represent low, moderate, and significant heterogeneity (14). All statistical analyses were conducted with the Cochrane Collaboration's Review Manager Software Package (RevMan 5.3).

## Results

### Study selection

**Figure 1:**
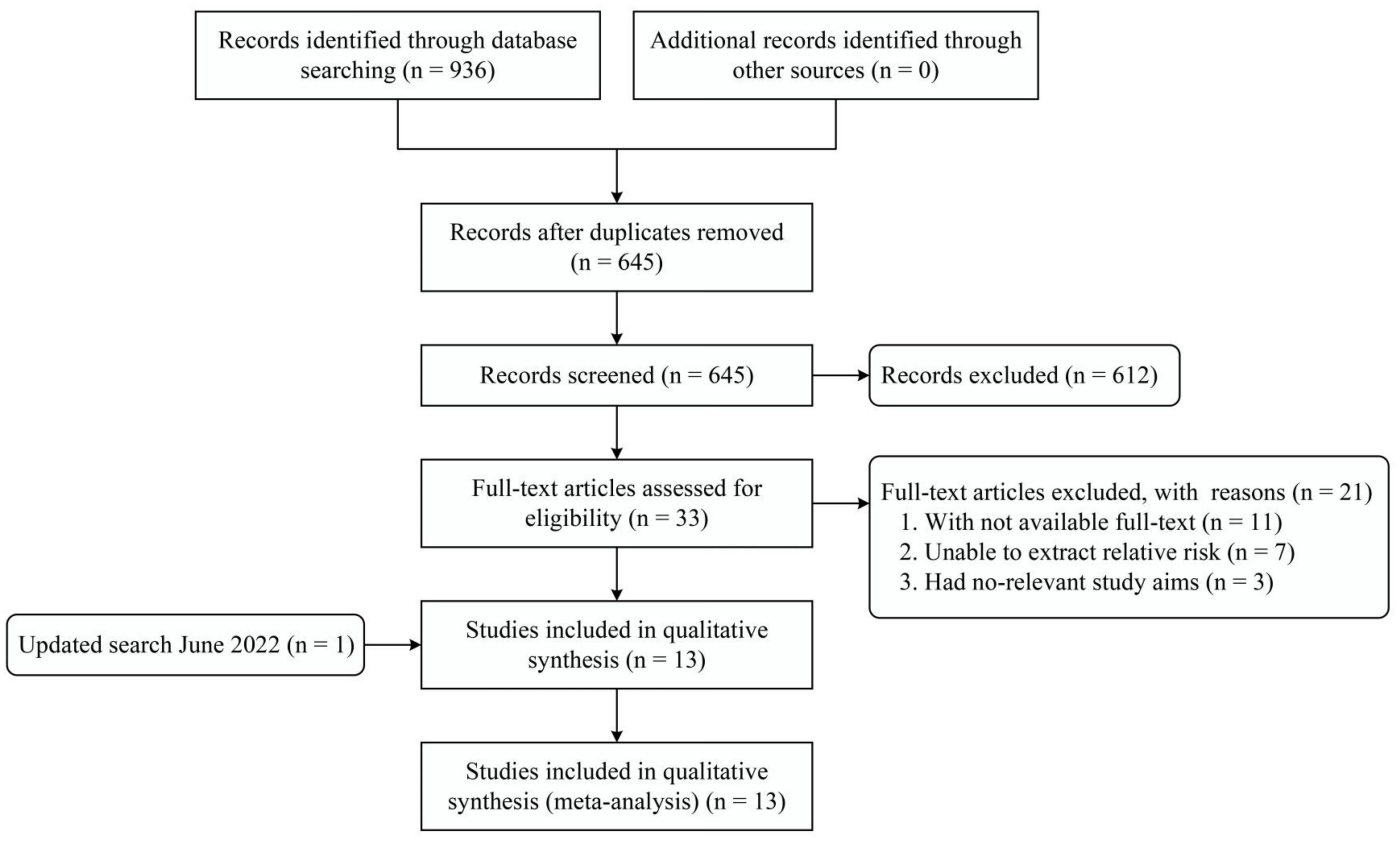
PRISMA flow diagram.

The initial search yielded 936 records, of which 291 records were repeats, and 612 records were excluded after reading the titles and abstracts. After reviewing the remaining 33 full-text articles, 12 articles (15–26) met the inclusion criteria. In addition, one relevant study (27) was identified from the updated search. Finally, 13 articles were included in this systematic review and meta-analysis. The screening process and reasons for exclusion are shown in Figure 1.

### Study characteristics and patient demographics

A total of 13 articles published between 2018 and 2022 were finally included for analysis. All these studies were retrospective observational studies based on prospective databases or prospective registries, and 10 of them were multi-centered. Overall, 2,271 patients underwent EVT, whereas 354 patients were treated with standard medical treatment. Eight studies reported the impact of EVT in a series of patients with prestroke disability stratified by prestroke mRS. Three studies compared the safety and efficacy of EVT and standard medical treatments for patients with pre-stroke disability. Five studies investigated whether successful recanalization would benefit patients treated with EVT. Age, sex, NIHSS, and ASPECTS were comparable for the intervention and control groups (*p* > 0.05). The characteristics of the included studies are summarized in Table 1.

**Table 1:** Characteristics of the studies included in the meta-analysis.

**Study**	**Study** **period**	**Country**	**Study** **design**	**No. of** **institution**	**Definition of** **PSD**	**No. of** **EVT**	**No. of** **SMT**	**Age** ^*^		**Male (%)**		**NIHSS** ^*^		**ASPECTS** ^*^	
								**Intervention**	**Control**	**Intervention**	**Control**	**Intervention**	**Control**	**Intervention**	**Control**
Goldhoorn et al. (15)	2014–2016	Netherlands	RO, PR	MR CLEAN	mRS 2–5	249	NA	80	NA	41	NA	17	NA	9	NA
Seker et al. (16)	2009–2017	Germany	RO, PD	Single	mRS 3–4	136	NA	NA	NA	NA	NA	NA	NA	NA	NA
Larsson et al. (17)	2015–2018	Sweden	RO, PR	SSR	mRS 2–4	161	NA	86	NA	40	NA	18	NA	NA	NA
Salwi et al. (18)	2015–2018	USA	RO, PD	Two stroke centers	mRS 2–3	243	NA	80	NA	37	NA	17	NA	9	NA
de Havenon et al. (19)	2013–2015	USA	RO, PR	TRACK	mRS 2–4	53	NA	73	NA	34	NA	18	NA	NA	NA
Florent et al. (20)	2015–2018	France	RO, PD	Single	mRS 3–5	155	NA	NA	NA	NA	NA	NA	NA	NA	NA
Kastrup et al. (21)	2008–2019	Germany	RO, PD	Single	mRS 3–4	142	89	83	86	NA	NA	17	15	8	8
Nababan et al. (22)	2016–2020	Australia	RO, PD	3 stroke centers	mRS 3	82	NA	85	NA	45	NA	17	NA	9	NA
Tanaka et al. (23)	2014–2016	Japan	RO, PR	RESCUE	mRS 2–4	175	164	82	87	35	25	19	22	7	7
Benali et al. (27)	2014–2017	Netherlands	RO, PR	MR CLEAN	mRS 3	190	NA	80	81	32	32	16	15	NA	NA
Ducroux et al. (24)	2016–2019	Canada and France	RO, PR	16 stroke centers	mRS 3–5	278	NA	81	78	30	39	19	20	8	9
McDonough et al. (25)	2010–2014	International	RO, PR	HERMES	mRS 1–2	98	101	70	73	NA	NA	18	17	8	8
Millán et al. (26)	2017–2019	Catalonia	RO, PR	CICAT	mRS 2–3	409	NA	77	NA	42	NA	17	NA	10	NA

* Data presented as mean or median.

ASPECTS, Alberta Stroke Program Early CT Score; CICAT, data from the Codi Ictus Catalunya Registry; EVT, endovascular thrombectomy; HERMES, data from the Highly Effective Reperfusion evaluated in Multiple Endovascular Stroke Trials collaboration; MR CLEAN, data from the Multicenter Randomized Clinical Trial of Endovascular Treatment of Acute Ischemic Stroke Registry; mRS, modified Rankin Scale; NA, Not available; NIHSS, National Institutes of Health Stroke Scale; PD, prospective database; PR, prospective registry; PSD, prestroke disability; RESCUE, data from the Recovery by Endovascular Salvage for Cerebral Ultra-Acute Embolism-Japan Registry 2; RO, retrospective observational; SMT, standard medical therapy; SSR, data from the Sahlgrenska Stroke Recanalization Registry; TRACK, data from the TREVO Stent-Retriever Acute Stroke Registry.

### Risk of bias assessment of included studies

The quality of all studies included in this systematic review and meta-analysis was considered reasonable. Of the 13 observational studies, six were rated as having a low risk of bias (good quality), and seven were rated as having a moderate risk of bias (fair quality). The results of the bias assessment are shown in Table 2.

**Table 2:** Results of quality assessment using the Newcastle–Ottawa Scale.

**Study**	**Questions of the quality assessment tool for cohort studies**								
	^**1**^	^**2**^	^**3**^	^**4**^	^**5**^	^**6**^	^**7**^	^**8**^	^**9**^
Goldhoorn et al. (15)	Yes	Yes	Yes	Yes	No	No	Yes	Yes	No
Seker et al. (16)	Yes	Yes	Yes	Yes	No	No	Yes	Yes	Yes
Larsson et al. (17)	Yes	Yes	Yes	Yes	No	No	Yes	Yes	Yes
Salwi et al. (18)	Yes	Yes	Yes	Yes	Yes	Yes	Yes	Yes	No
de Havenon et al. (19)	Yes	Yes	Yes	Yes	Yes	Yes	Yes	Yes	No
Florent et al. (20)	Yes	Yes	Yes	Yes	Yes	Yes	Yes	Yes	Yes
Kastrup et al. (21)	Yes	Yes	Yes	Yes	No	No	Yes	Yes	Yes
Nababan et al. (22)	Yes	Yes	Yes	Yes	No	No	Yes	Yes	Yes
Tanaka et al. (23)	Yes	Yes	Yes	Yes	Yes	Yes	Yes	Yes	Yes
Benali et al. (27)	Yes	Yes	Yes	Yes	Yes	Yes	Yes	Yes	Yes
Ducroux et al. (24)	Yes	Yes	Yes	Yes	No	No	Yes	Yes	No
McDonough et al. (25)	No	Yes	Yes	Yes	No	No	Yes	Yes	Yes
Millán et al. (26)	Yes	Yes	Yes	Yes	Yes	Yes	Yes	Yes	No

1 Is the exposed cohort truly representative of the average population in the community?

2 Was the non-exposed cohort drawn from the same community as the exposed cohort?

3 Was the ascertainment of exposure from a secure record?

4 Was it demonstrated that the outcome of interest was not present at the start of the study?

5 Comparability of cohorts on the basis of the design or analysis: study controls for age and sex?

6 Comparability of cohorts on the basis of the design or analysis: study additionally controls for other factors?

7 Was the assessment of outcome performed by record linkage?

8 Was the follow-up long enough for outcomes to occur?

9 Was the follow-up of the cohorts adequate?

Green stands for yes, red for no.

### Clinical outcomes after endovascular thrombectomy and medical treatment in patients with prestroke disability

#### Proportion meta-analysis

**Figure 2:**
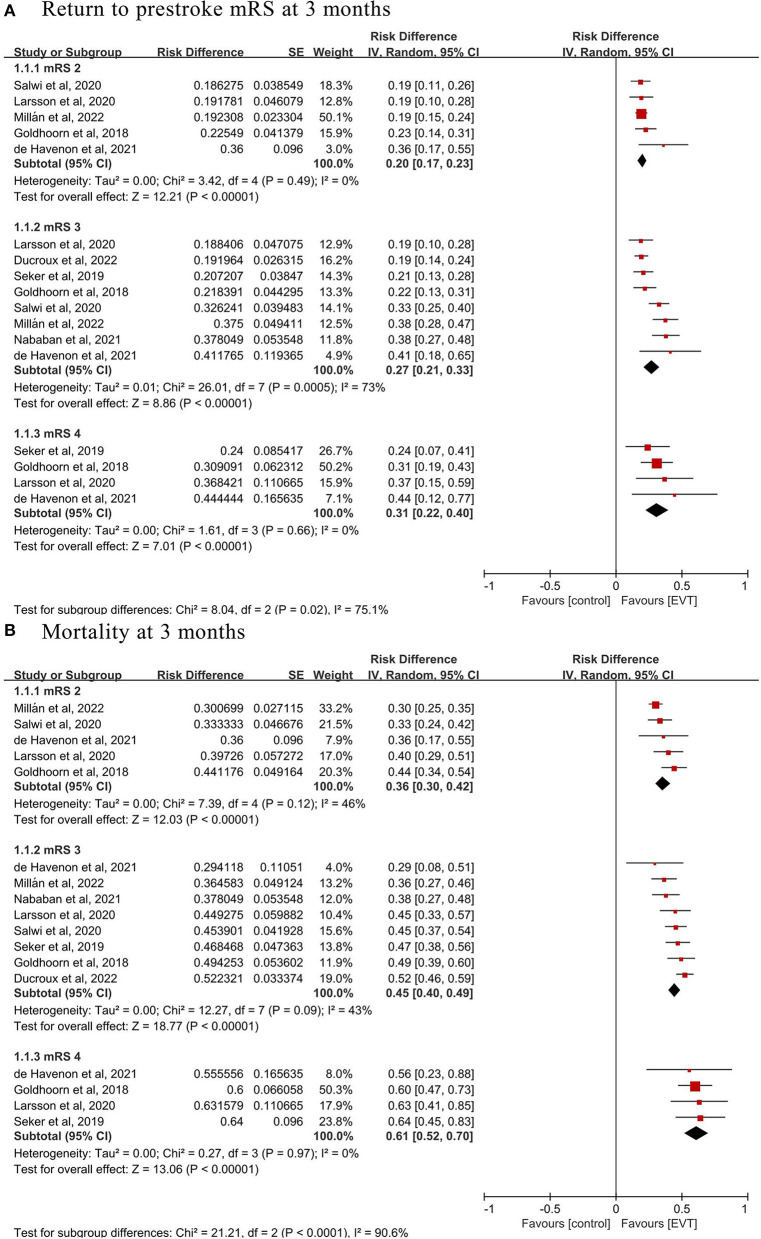
Pooled incidence of **(A)** return to prestroke modified Rankin Scale (mRS) and **(B)** mortality at 3 months in prestroke disability patients treated with endovascular thrombectomy (EVT).

Eight of the 13 studies, including 1,530 patients with prestroke disability treated with EVT, reported the distribution of mRS shifts and death at 3 months by prestroke mRS. The overall rate of return to prestroke mRS at 3 months was 20% (mRS = 2; 120/588, 95% CI 17 to 23%, *I*^2^ = 0%; Figure 2 and Table 3), 27% (mRS = 3; 218/827, 95% CI 21 to 33%, *I*^2^ = 73%; Figure 2 and Table 3), and 31% (mRS = 4; 34/108, 95% CI 22 to 40%, *I*^2^=0%; Figure 2 and Table 3), respectively, after EVT. There were only seven patients in the group with prestroke mRS of five, and the overall rate of return to prestroke mRS was 29%. The rate of mortality at 3 months after EVT was 36% (mRS = 2; 203/588, 95% CI 30 to 42%, *I*^2^ = 46%; Figure 2 and Table 3), 45% (mRS = 3; 378/827, 95% CI 40 to 49%, *I*^2^ = 43%; Figure 2 and Table 3), and 61% (mRS = 4; 66/108, 95% CI 52 to 70%, *I*^2^ = 0%; Figure 2 and Table 3), respectively.

**Table 3:** Pooled outcomes.

**Variables**	**Studies (*n*)**	**Intervention (*n*)**	**Control (*n*)**	**Effect size (95% CI)**	***P*** **-value**	***I*** **^2^**
**Clinical outcomes among EVT**						
**Return to prestroke mRS**						
mRS = 2 group	5	588	NA	0.20 (0.17–0.23)	-	0%
mRS = 3 group	8	827	NA	0.27 (0.21–0.33)	-	73%
mRS = 4 group	4	108	NA	0.31 (0.22–0.40)	-	0%
mRS = 5 group	2	7	NA	2/7^*^	-	-
**Mortality**						
mRS = 2 group	5	588	NA	0.36 (0.30–0.42)	-	46%
mRS = 3 group	8	827	NA	0.45 (0.40–0.49)	-	43%
mRS = 4 group	4	108	NA	0.61 (0.52–0.70)	-	0%
mRS = 5 group	2	7	NA	5/7^*^	-	-
**Comparison between EVT group and standard medical treatment group**						
Return to prestroke mRS	3	416	354	1.86 (1.28–2.70)	0.001	53%
Mortality	3	416	354	0.75 (0.58–0.97)	0.03	0%
**Comparison between successful recanalization vs. no recanalization after EVT**						
Return to prestroke mRS	4	508	189	2.04 (1.17–3.55)	0.01	50%
Mortality	3	431	180	0.72 (0.62–0.84)	<0.001	0%

* Data presented as events/total.

EVT, endovascular thrombectomy; mRS, modified Rankin Scale; NA, Not available.

#### Comparative meta-analysis

**Figure 3:**
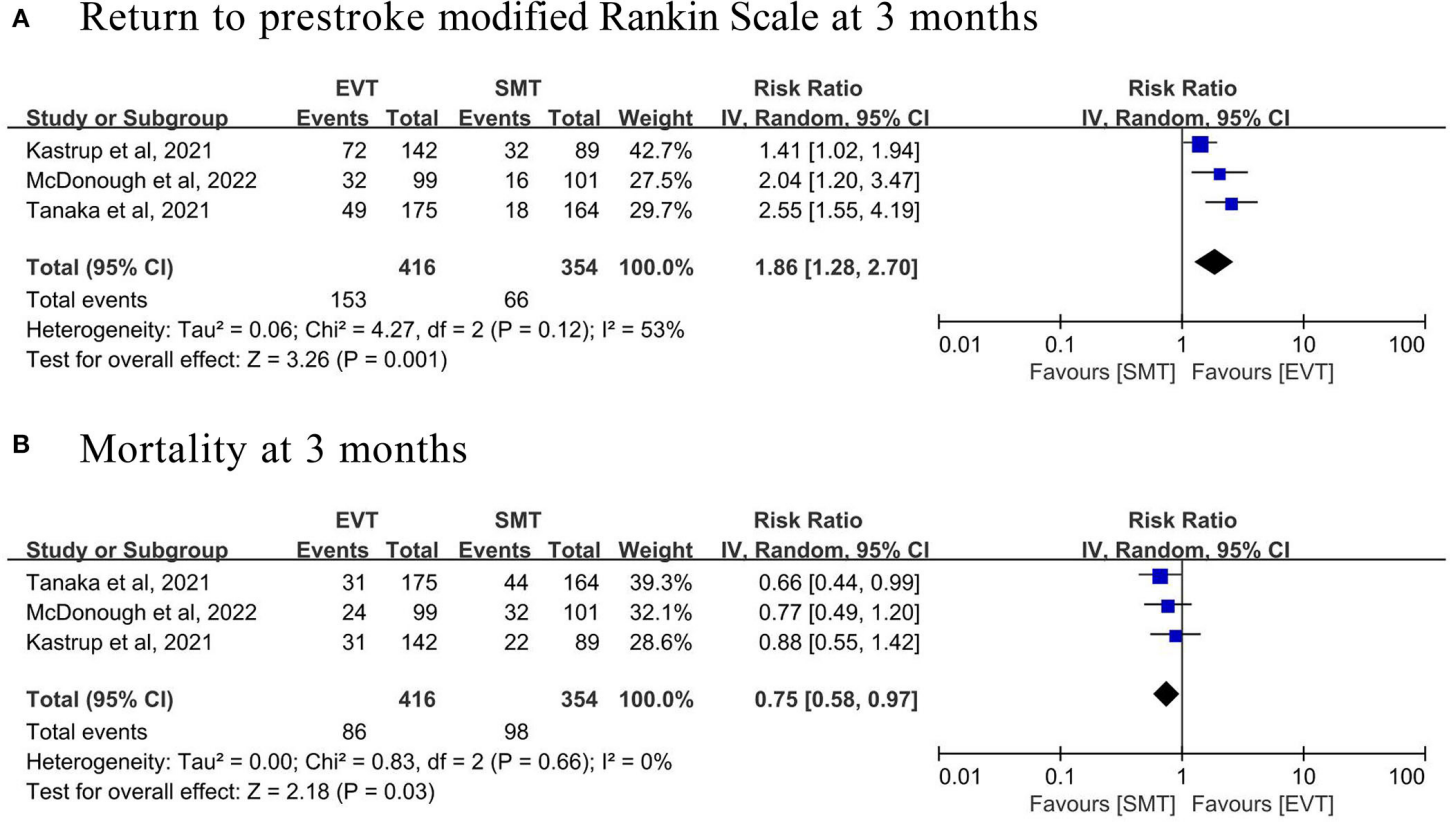
Forest plot of **(A)** return to prestroke modified Rankin Scale and **(B)** mortality at 3 months in prestroke disability patients treated with endovascular thrombectomy (EVT) vs. standard medical therapy (SMT).

Three of the 13 studies, involving 769 patients (415 EVT and 354 standard medical treatments), compared the safety and efficacy between EVT and standard medical treatments for patients with prestroke disability, which were selected for the comparative meta-analysis. The pooled analysis found that EVT was related to a higher likelihood of return to prestroke mRS at 3 months (three studies; RR 1.86, 95% CI 1.28–2.70, *p* = 0.001, *I*^2^ = 53%; Figure 3 and Table 3) and a lower likelihood of mortality at 3 months (three studies; RR 0.75, 95% CI 0.58–0.97, *p* = 0.03, *I*^2^ = 0%; Figure 3 and Table 3) compared with standard medical treatments.

**Figure 4:**
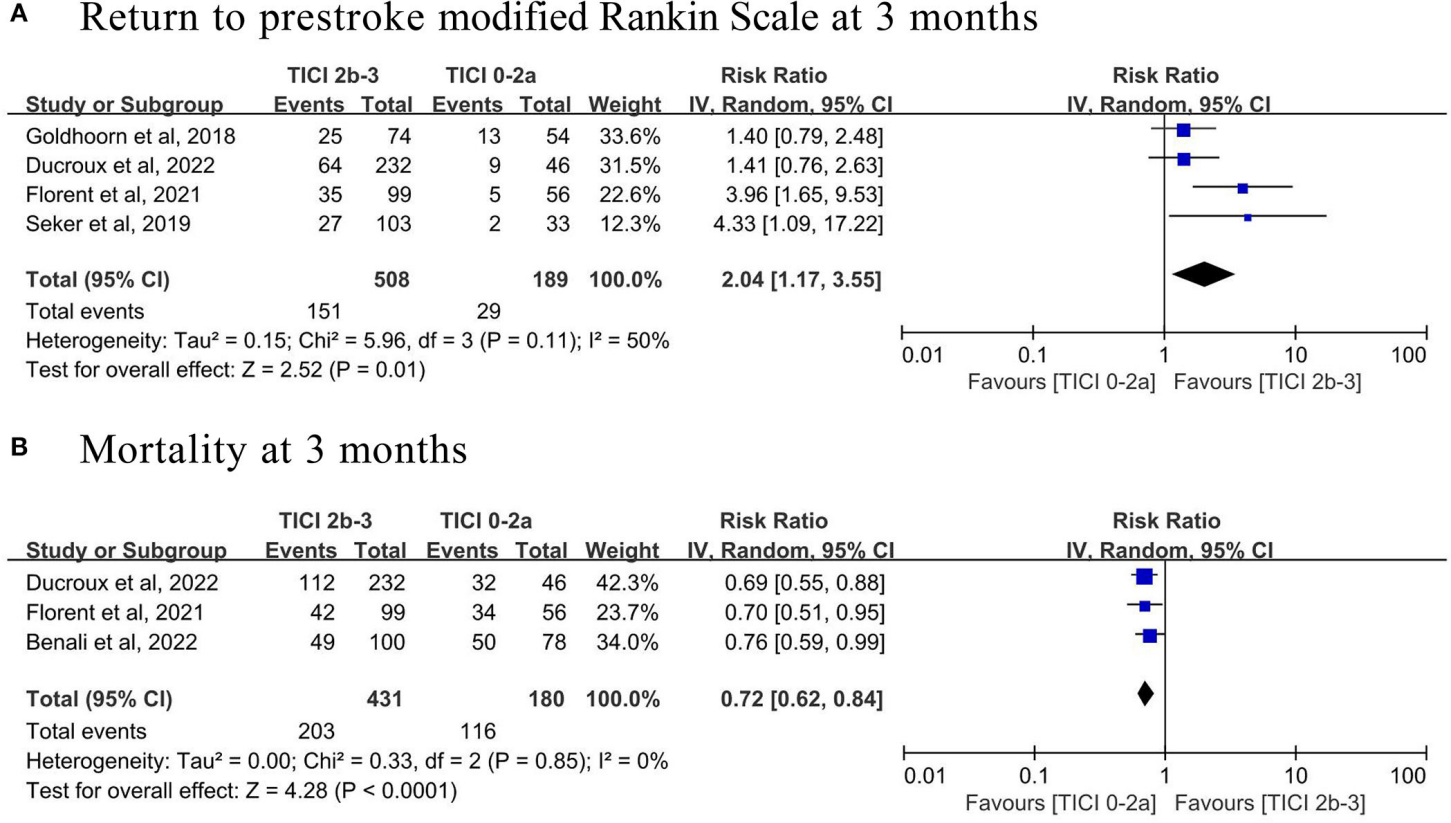
Forest plot of **(A)** return to prestroke modified Rankin Scale and **(B)** mortality at 3 months in prestroke disability patients with vs. without successful recanalization (Thrombolysis In Cerebral Ischemia (TICI) 2b-3) after endovascular thrombectomy.

Five of the 13 studies, involving 887 patients (616 with successful recanalization and 271 with no recanalization), analyzed whether successful recanalization benefited patients with prestroke disability undergoing EVT, which were selected for the comparative meta-analysis. The pooled analysis found that patients with successful recanalization achieved by EVT had a higher proportion of return to prestroke mRS at 3 months (four studies; RR 2.04, 95% CI 1.17–3.55, *p* = 0.01, *I*^2^ = 50%; Figure 4 and Table 3) and lower mortality at 3 months (three studies; RR 0.72, 95% CI 0.62–0.84, *p* < 0.001, *I*^2^ = 0%; Figure 4 and Table 3), compared with patients without successful recanalization.

## Discussion

Our meta-analysis of nearly 3,000 acute ischemic stroke patients with prestroke mRS ≥2 showed that (1) the chance of a return to prestroke mRS at 3 months was slightly increased with increasing prestroke mRS and significantly increased mortality was found at 3 months; (2) as compared with patients treated with standard medical treatments, EVT increased the rate of favorable clinical outcomes; and (3) patients with successful reperfusion after EVT had a higher likelihood of returning to prestroke mRS and lower risk of mortality compared with those having no reperfusion.

In general, clinicians do not always consider EVT for eligible ischemic stroke patients with prestroke disability due to the fact that they were more likely to die (6, 28). Our findings were in line with previous studies (28) that showed every point increase in prestroke mRS was associated with a higher risk of mortality (28). However, this association was not explained by the influence of prestroke disability on EVT. The higher mortality would probably be related to prestroke comorbidity and frailty and medical complexity (29, 30). Surprisingly, prestroke disability patients treated with EVT had a higher chance of returning to prestroke mRS with increasing prestroke mRS. Indeed, using typical dichotomy-based definitions of favorable outcomes (e.g., mRS, 0–1, or 0–2) did not show significance in patients with prestroke disability, as it set an unattainable and unjust bar of success. Thus, it may be reasonable for patients with prestroke disability to maintain their premorbid status to be considered favorable outcomes. Although the above results cannot be ascribed to the interaction of prestroke disability and the effects of EVT, there was no association between prestroke mRS and accumulated disability. These data supported a more inclusive EVT selection paradigm with regard to prestroke disability. More importantly, the results of this meta-analysis were in line with other series concerning patients with mRS of 0–1 treated endovascularly (31, 32), which showed that EVT for patients with prestroke disability gave a higher rate of return to prestroke mRS and lower mortality at 3-month follow-up compared with medical management. Our analyses also suggested that successful reperfusion after EVT resulted in a higher likelihood of return to prestroke mRS and a lower risk of mortality compared with those having no reperfusion, in line with evidence suggesting that patients with larger infarct cores may benefit from EVT (33). EVT should be recommended on the basis of evidence from comparative meta-analysis and be considered an effective treatment for acute ischemic stroke.

Current guidelines from the Chinese Stroke Association (1) and the American Heart Association/American Stroke Association (2) suggest that EVT may be reasonable for acute ischemic stroke patients with prestroke mRS ≥2 (class IIb, level of evidence B), whereas the European Stroke Organization (3) does not mention prestroke disability in their guidelines on EVT in acute ischemic stroke due to a lack of trial evidence. We hoped the presented data would prompt the guideline to add a statement that prestroke disability should not be regarded as an exclusion criterion for EVT treatment. Future randomized controlled trials are still needed to validate the efficacy and safety of EVT in stroke patients with prestroke disability. Further studies of deaths and health and social care costs in acute ischemic stroke patients with prestroke disability treated with EVT would also be meaningful, stratified by baseline mRS.

The outcome data, references, and subgroup analyses included in the present meta-analysis were more comprehensive and complete, containing a larger sample size, than those previously published meta-analyses. However, several irresolvable limitations of the present report need to be acknowledged. First, all of the studies included herein were observational, which were susceptible to biases, and limited the validity of our findings. Second, publication bias across individual studies was not evaluated because the recommended minimum of 10 studies per outcome was not met. Third, we could not draw valid conclusions about patients with a prestroke mRS of 5 because EVT was very uncommon in this population. Four, one (25) of the three studies that compared the outcomes between EVT and SMT involved some patients with mRS 1, also limiting the validity of our findings.

## Conclusion

The present meta-analysis found that increased prestroke mRS in acute ischemic stroke patients had no association with accumulated disability, despite a higher probability of death. For eligible ischemic stroke patients with prestroke disability, EVT significantly improved clinical outcomes compared with standard medical treatments alone. Successful recanalization increased the probability of return to the prestroke level of disability and lower mortality. These findings show that prestroke disability should not be regarded as an exclusion criterion from EVT practice.

## Data availability statement

The original contributions presented in the study are included in the article/supplementary material, further inquiries can be directed to the corresponding author/s.

## Author contributions

J-CY and Q-JB: conception and design of the study, acquisition of data, analysis and interpretation of data, and drafting of the article. YG: acquisition of data, analysis and interpretation of data, and drafting of the article. S-JC and J-TZ: acquisition of data and analysis and interpretation of data. QZ and PZ: revising the manuscript critically for important intellectual content. M-FY: the conception and design of the study and revising the manuscript critically for important intellectual content. All authors have read and approved the final version of the manuscript.

## Funding

This study was supported by the Science and Technology Department of Qinghai Province (No. 2020-ZJ-774).

## Conflict of interest

The authors declare that the research was conducted in the absence of any commercial or financial relationships that could be construed as a potential conflict of interest.

## Publisher's note

All claims expressed in this article are solely those of the authors and do not necessarily represent those of their affiliated organizations, or those of the publisher, the editors and the reviewers. Any product that may be evaluated in this article, or claim that may be made by its manufacturer, is not guaranteed or endorsed by the publisher.
